# Food Consumption Pattern and the Intake of Sugar, Salt, and Fat in the South Jakarta City—Indonesia

**DOI:** 10.3390/nu13041289

**Published:** 2021-04-14

**Authors:** Nuri Andarwulan, Siti Madanijah, Dodik Briawan, Khoirul Anwar, Atikah Bararah, Saraswati, Dominika Średnicka-Tober

**Affiliations:** 1 Department of Food Science and Technology, Faculty of Agricultural Technology, IPB University, P.O. Box 220, IPB Darmaga Campus, Bogor 16680, West Java, Indonesia; atikah.bararah@gmail.com (A.B.); saraswati_231@apps.ipb.ac.id (S.); 2 Southeast Asian Food and Agricultural Science and Technology (SEAFAST) Center, IPB University, IPB Darmaga Campus, Bogor 16680, West Java, Indonesia; sitima@apps.ipb.ac.id (S.M.); dbriawan@apps.ipb.ac.id (D.B.); 3 Department of Community Nutrition, Faculty of Human Ecology, IPB University, P.O. Box 220, IPB Darmaga Campus, Bogor 16680, West Java, Indonesia; khoirul_anwar@usahid.ac.id; 4 Department of Functional and Organic Food, Institute of Human Nutrition Sciences, Warsaw University of Life Sciences, Nowoursynowska 159c, 02-776 Warsaw, Poland

**Keywords:** fat, food record, non-communicable diseases, salt, sugar, South Jakarta, Indonesia

## Abstract

The excessive consumption of sugar, salt, and fat is associated with an increased risk of non-communicable diseases. Therefore, a study on estimating the added sugar, salt, and fat intake in certain populations is important for establishing specific recommendations aiming at improving diet quality, and thus public health. This study aimed to determine the food consumption pattern and the intakes of added sugar, salt, and fat from different food groups and food sources among the residents of South Jakarta, Indonesia. The study was conducted with a cross-sectional design, involving 323 respondents. Data on socio-economic conditions, health and nutritional status, and food consumption were collected. Food consumption data were acquired through the 2-day weighed food record. Results showed that the daily food consumption in the observed population reached 1868–2334 g/capita/day. The total added sugar intake in different groups of respondents ranged between 34.9 and 45.9 g/capita/day, with the highest values observed in school-age boys. Beverages and snacks were identified as the main added sugar sources in the respondents’ diet. The total salt intake ranged from 5.46 to 7.43 g/capita/day, while the observed fat intake reached 49.0–65.1 g/capita/day. The major food source contributing to the salt and fat intake included street/restaurant/fast food. Male subjects tended to consume a higher amount of salt and fat than female subjects. These findings can be used as baseline information for providing a strategy for reducing sugar, salt, and fat intakes, with strong implications for improving public health.

## 1. Introduction

Non-communicable diseases (NCDs), also known as chronic diseases, are reported to cause the death of 41 million people each year, equivalent to 71% of all deaths globally. NCDs usually occur around the age of 30–69, and over 85% of these “premature” deaths occur in low- and middle-income countries. The main types of NCDs are cardiovascular diseases (e.g., stroke and heart attacks), cancers, chronic respiratory diseases, and diabetes mellitus. The risk of dying from NCDs is strongly associated with physical inactivity, tobacco use, use of alcohol, and unhealthy diets [1]. In Indonesia, these diseases are also identified as the major cause of death. Currently, the prevalence of NCDs in Indonesia, monitored based on the National Basic Health Research 2007, 2013, and 2018, is increasingly worrying [2,3,4]. This is due to the fact that the increasing trend of NCDs is followed by a shift in disease patterns. In the past, these types of diseases were usually experienced by the elderly, while now they are starting to threaten the productive age group. The prevalence of cancers, stroke, chronic kidney disease, diabetes mellitus, and hypertension in Indonesia is reported to increase from year to year, also in the younger age groups [5].

Poor diets are an important preventable risk factor for NCDs. A number of epidemiological and case-control studies reported the associations between excessive dietary sugars, salt, and fat and increased risk of NCDs, including obesity, hypertension, diabetes, cardiovascular diseases, and certain cancers [6]. In addition, diets low in vegetables, fruits, legumes, whole grain, nuts and seeds, milk, and omega-3 polyunsaturated fatty acids are identified as the other NCDs risk factors [7]. Actually, salt, sugar, and fat had been important elements of human diet, both in terms of their role in the body, and their ability to build a savory, sweet, and pleasant taste of food [6]. However, considering the potential of an excessive intake of these three elements to cause adverse health effects, controlling the consumption of salt, sugar, and fat becomes very important. These efforts can be started by evaluating the consumption level in certain populations. Studies on the total added sugar, salt, and fat intakes in Indonesia had been reported by several research groups [8,9,10,11,12,13]. However, information regarding the contribution of food sources/origin (homemade, processed or street/restaurant/fast foods) to the daily food consumption, including sugar, salt, and fat intakes, has not previously been reported in the Indonesian population. Detailed information regarding food consumption patterns and the contribution of food types/groups and food sources to sugar, salt, and fat intakes are needed for setting realistic goals and plans for interventions to reduce the consumption of those nutrients.

## 2. Materials and Methods

### 2.1. Study Design

This was a cross sectional study, conducted for 2 months in Rawajati Village of Pancoran Sub-district, South Jakarta (Special Capital Region of Jakarta Province). The study received the ethical approval from the Health Research Ethics Commission, Faculty of Medicine, Diponegoro University (protocol no.: 24/EC/FK-RSDK/2015). The general population was classified into three different age groups (children, adolescents, and adults). The minimum sample size required for each group was determined based on Cochran’s formula for the proportional variable as also used in the study of Anisyah et al. [14]. According to that method, each demographic group should be represented by at least 50 respondents. The sample size was calculated according to the following formula:

*n* = (Z_α/2_^2^ × p × q)/E^2^ (1)

Note: *n* = sample size; Z = 1.96 (the value was obtained from the normal distribution table at the confidence level of 95%); α = significance level; p = population proportion (assumed to be 0.5 since it was unknown); q = 1 − p; E = margin of error (assumed to be 14%).

The studied population included more than 100 households, which specifically had family members representing three different age groups: School-age children (6–12 years), adolescents (13–18 years), and adults (≥19 years). Thus, the total number of subjects was 323. The selection of subjects was purposively carried out by considering several characteristics, such as socio-economic status (including income and occupation), education level, and gender. The following additional inclusion criteria related to health status and diet were also implemented: Only healthy subjects, not suffering from NCDs such as i.e., diabetes, high blood pressure, and heart disease (as declared by the respondents, based on their knowledge) and not undergoing a certain diet program, were included in the study. Table 1 shows a number of study subjects based on gender and age group.

### 2.2. Data Collection

The data on food consumption pattern and subjects’ characteristics (i.e., health status, body height and weight, level of education, income, occupation, gender, and age) were collected in this study. Subjects’ characteristics data, such as health status, education level, income, occupation, gender, and age, were recorded through interviews using a structured questionnaire. The body height, weight, and blood pressure were directly measured by trained project staff during the interview, while food consumption patterns were recapitulated using the weighed food record method for 2 days (weekday and weekend). Trained project staff conducted home visits during the study period to ensure proper recording and weighing procedures. All questionnaires were checked for the completeness and errors in the same day or the day after being administered. Missing data were collected by revisiting the respondents. This 2-day weighed food record method had previously been used by several research groups [15,16,17] as a reference method for assessing dietary intake. This food consumption survey was also carried out in accordance with the guidelines of FAO [18].

To obtain data on sugar, salt, and fat added to homemade foods/dishes, a food record was also carried out at the household level, together with the individual subject’s food record. The household food record was filled out by a person responsible for providing food in the household. The household food record allowed documenting detailed information about food ingredients used to produce homemade food, including their respective weights. At the same time, individual food records were filled out by each respondent, and verification by older siblings/parents was also carried out for food record data of school-age respondents.

### 2.3. Estimation of Added Sugar, Salt, and Fat Intake

Food consumption data obtained from 2-day weighed food records were grouped based on two categories, namely the food group and food sources. Foods were classified into thirteen food groups, i.e., bakery products, beverages, cereals and cereal products, eggs and egg products, fish and fish products, fruits and fruit products, legumes and legume products, meat and meat products, milk and dairy products, chicken and poultry products, snacks, vegetables and vegetable products, and supplements. All food items were also categorized into three different sources: Processed foods, homemade foods, and street/restaurant/fast foods.

Food consumption data were expressed in grams. The sugar, salt, and fat content of foods was determined by referring to various databases, such as The Indonesian Food Composition Table 2005 [19], The USDA National Nutrient Database for Standard Reference 2015 [20], and The Food Composition Table of Singapore [21].

#### 2.3.1. Estimation of Added Sugar Intake

The term “sugar” in this study was referred to monosaccharides and disaccharides added to food during processing and serving (brown sugar and/or white sugar), as well as sugar present in honey, syrups, fruit juices, and concentrates. The amount of sugar added (total sugar) in the processed foods was directly obtained from the food labels (commercial products enlisted by the National Agency for Drug and Food Control—Indonesia), while the amount of total added sugar in the homemade food and street/restaurant/fast food was determined by several ways, i.e., (a) recording the amount of sugar added when cooking or serving food, (b) recording the amount of sugar added based on the food recipe elaboration, both as a result of the interview session and browsing on the trusted recipe websites, (c) referring to The Food Composition Tables [19,21] and USDA reference data [19], (d) if there were no detailed recipes provided, the laboratory analysis was carried out with a digital refractometer (SCM1000, HM Digital, Redondo Beach, CA, USA) which had been calibrated by the Anthrone method for sugar analysis (the R^2^ of those tested methods was 0.99).

The food sample for the digital refractometer analysis was firstly prepared as also performed in the Anthrone method described by Pomeranz and Meloan [22]. The prepared sample was placed on the sample well of the refractometer. The refractometer reading was then taken. For each analysis, the prism was calibrated with distilled water. The sugar content (% Brix) was calculated by multiplying the sugar content read on the refractometer by the dilution factor. Then, the sugar content value which was used as a reference for calculating the sugar intake was the sugar content that had been converted with the equation of y = 1.027x +2.670, where x is the sugar content read using a digital refractometer and y is the actual sugar content value. This equation is generated from the correlation results of sugar contents obtained by the spectrophotometric method (Anthrone method) and the refractometric method. The comparison and correlation between the two methods had been previously established by the Southeast Asian Food and Agricultural Science and Technology (SEAFAST) Center. The added sugar intake was calculated as follows:

Added sugar intake (g) = added sugar content in food (g/g) × amount of food consumed (g)(2)

#### 2.3.2. Estimation of Salt Intake

The term “salt” in this study was the salt added in the form of NaCl when cooking, processing, and serving food. The sodium content (Na/g food) of the processed foods was obtained from the food labels (commercial products enlisted by the National Agency for Drug and Food Control—Indonesia), and the NaCl content was then calculated by considering the mole conversion factor. The determination of salt content in homemade food and street/restaurant/fast food followed several ways which were described in the section above for sugar, but the salt analysis for unavailable recipe information was conducted using a salt check meter (SB-2000Pro, HM Digital, Redondo Beach, CA, USA) which had been calibrated by the argentometric titration method (Mohr method) as described in the AOAC Official Method 960.29 [23] (the R^2^ of those two methods was 0.94).

The determination of salt content for liquid samples using a salt check meter can be done directly. As for the solid sample, about 1 g of the sample was firstly dissolved in hot water and homogenized using a magnetic stirrer for 5–10 min until the temperature of the sample solution was around 50–55 °C. The solution was subsequently allowed to stand at room temperature, then the salt content of the solution was measured using a salt check meter. The salt content (%) was calculated by multiplying the read value on the instrument by the dilution factor. The total NaCl and Na intake was calculated as follows:

NaCl intake (g) = NaCl content in food (g/g) × amount of food consumed (g)(3)

Na intake (mg) = (NaCl intake (g) × 1000)/2.5(4)

#### 2.3.3. Estimation of Fat Intake

The total fat content of the processed foods was obtained from the food labels (commercial products enlisted by the National Agency for Drug and Food Control—Indonesia), while the determination of total fat content of homemade food and street/restaurant/fast food was performed by referring to The Food Composition Tables [19,21], The USDA reference data [20], elaboration of food recipes, and reference database of fat absorption of foodstuffs processed by frying. The total fat intake was calculated as follows (4):

Fat intake (g) = fat content in food (g/g) × amount of food consumed (g)(5)

### 2.4. Data Analysis

**Figure 1:**
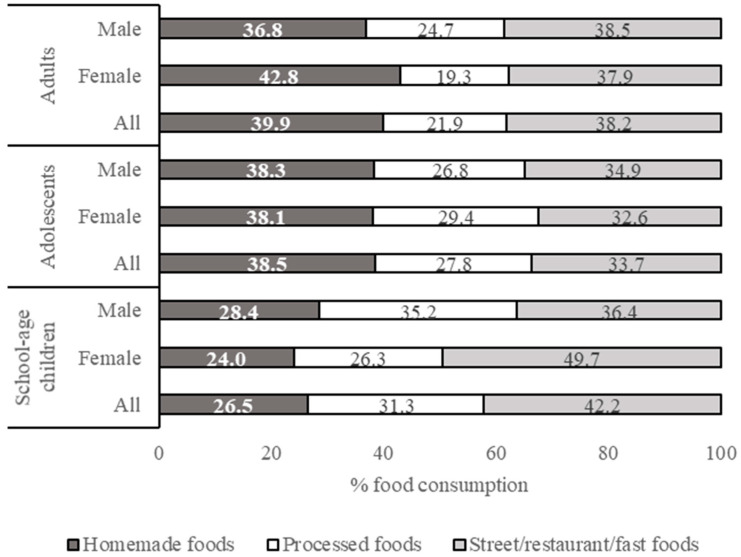
Contribution of different food sources to the total food consumption.

**Figure 2:**
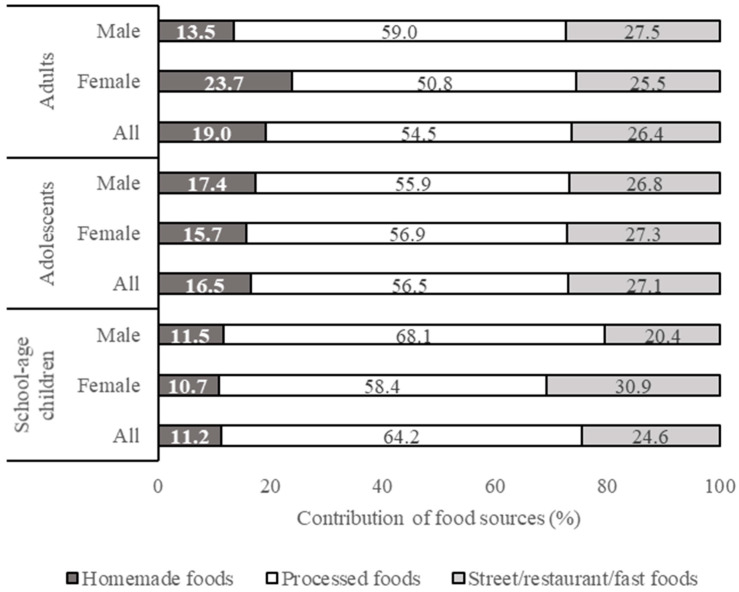
Contribution of different food sources to the total added sugar intake.

**Figure 3:**
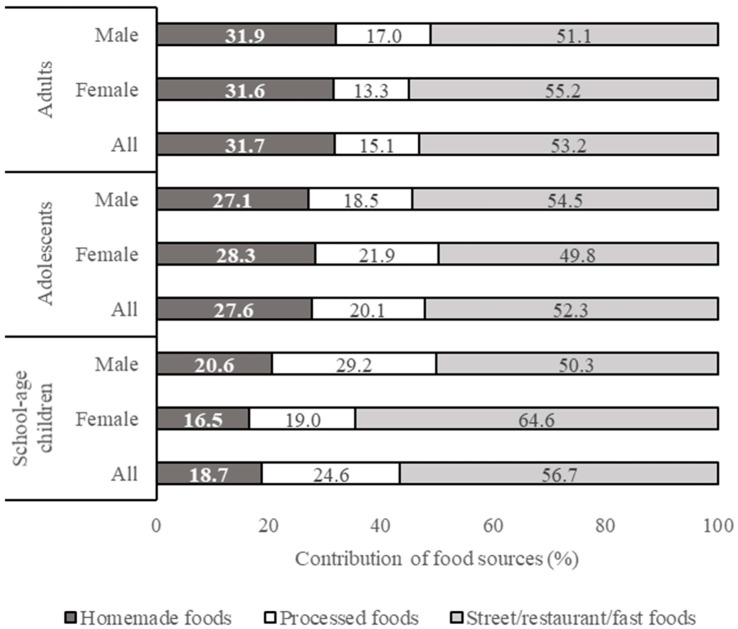
Contribution of different food sources to the total salt intake.

**Figure 4:**
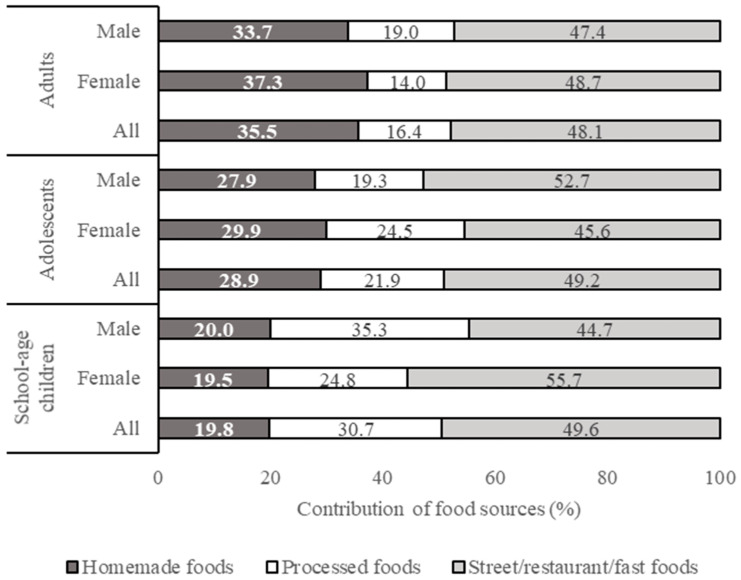
Contribution of different food sources to the total fat intake.

Results on subject characteristics, health and nutritional status, and food consumption pattern were processed using Microsoft Excel 2013 and expressed descriptively. The results on the health and nutritional status of respondents are presented in Tables 2 and 3 (mean % values or mean ± SD). The results on food intake from different food groups and food sources are presented in Table 4 (mean ± SD, in g/capita/day) and in Figure 1 (percentage contribution of different food sources to total food intake), respectively. Details of the contribution of each food group to the daily calorie intake (kcal/cap/day) are shown in Table S1 (Supplementary Materials). The results on added sugar, salt, and fat intakes from different food groups and food sources are presented in Tables 6–8 (mean, minimum, and maximum, in g/capita/day) and in Figures 2–4 (percentage contribution of different food sources to total sugar, salt, and fat intake), respectively. The differences in total calorie, added sugar, salt, and fat intake between (a) different age groups and (b) male and female respondents within each age group were analyzed statistically using the independent samples *t*-test. Data homogeneity were tested using Levene’s test. Normality of the data was tested using Kolmogorov-Smirnov and Jarque-Bera’s tests. All statistical analyses were performed in the SPSS PASW v. 18 software (SPSS Inc., Chicago, IL, USA).

## 3. Results and Discussion

### 3.1. Subjects Characteristics

A total of 323 individual subjects with a proportional number of adults (male and female), adolescents (male and female), and school-age children (male and female) were observed based on various parameters, including socio-economic characteristics, health and nutritional status, and food consumption. Adult male respondents (*n* = 47) were dominated by private employees (63.8%) followed by government employees (10.6%), most of them with a senior high school (57.4%) and diploma/bachelor (29.8%) education. In addition, 72.4% of adult female respondents were housewives (not working outside) and 17.2% were self-employed/entrepreneur. The educational background of adult female respondents was also dominated by an education equivalent to senior high school (58.6%), and then followed by diploma/bachelor (27.6%). Overall, the education level of adult male and female respondents (*n* = 105) was dominated by senior high school equivalent (58.1%) and diploma/bachelor (28.6%).

Data on the health and nutritional status of respondents are presented in Table 2 and Table 3. The categorization of health and nutritional status was based on several references, as presented in the Tables’ footnotes [24,25,26,27]. Generally, 48.6% of adult respondents had a normal weight, 36.2% of them were categorized as overweight, and 9.5% were obese. The average BMI values of adult male and female respondents were 24.2 and 24.9, respectively. At the same time, 65.5% of adult females had the waist to hip ratio (WHR) above 0.8, which indicated the increased health risk. In addition, 70.2% of adult males were categorized to be at risk, since their WHR values were higher than the normal standard (≥0.95).

Although the declared health status (lack of NCDs) was one of the inclusion criteria in this study, diabetes mellitus and hypertension were identified in 1.9% and 3.8% of adult respondents, respectively. At the same time, less than 40% of the respondents’ family members had a history of the aforementioned diseases or cardiovascular disease.

The adolescent respondents were mainly from medium families (consisting of 5–7 family members) (48.6%) and small families (<5 family members) (37.4%), and their family incomes were mainly lower than IDR 5 million (50.5%) or between IDR 5 and 10 million (27.1%). Most of the adolescent respondents had normal height (94.4%) and normal BMI (75.7%). Four out of 51 male respondents showed the pre-hypertension indication.

In accordance with the findings on adolescents, school-age children also generally came from medium (55.9%) and small (44.1%) families, and their family incomes were mainly lower than IDR 5 million (57.7%) or between 5 and 10 million (35.1%). Nearly 90% of school-age children had normal height. There was one school-age girl identified to be severely stunted (z-score below −3). The majority of school-age respondents were found to have normal BMI. In addition, there were two school-age children showing pre-hypertension indication.

### 3.2. Food Consumption Pattern

Table 4 shows the food consumption pattern of all respondents. The daily food consumption of adults, adolescents, and school-age respondents was estimated as 2257, 2084, and 2028 g per capita per day, respectively. Generally, beverages, cereals, and cereal products, and vegetable-based foods were observed as the main contributors to the daily food consumption of all respondents. In addition, adolescent and school-age respondents consumed a greater amount of milk and dairy products than adult respondents, and the contribution of this food group to the total food consumption reached 4.46–8.07%.

Eggs, chicken, and poultry products have been identified as the major sources of animal protein for all respondents (3.6–5.03% of total food consumption), followed by fish and meat. Adult respondents consumed more legumes and legume products (2.33% of total food consumption) than the other age groups (1.24% for adolescents and 1.38% for school-age children). Adult respondents also consumed higher amounts of fruits and fruit products compared to the other respondents’ groups (2.85% of total food consumption), with male respondents found to consume higher amounts of those products than female respondents.

The observed mean calorie intakes (kcal/capita/day) were 1702 ± 533 for adults, 1762 ± 586 for adolescents, and 1705 ± 551 for school-age children group. Details of the contribution of each food group to the daily calorie intake can be seen in Table S1 (Supplementary Materials). In general, male respondents tended to get a higher calorie intake than women. Age groups did not appear to differ significantly in the calorie intake. In accordance with the level of consumption, cereals and cereal products (742–897 kcal/cap/day) were the main contributors to the calorie intake. Furthermore, snacks (205–263 kcal/cap/day), beverages (119–187 kcal/cap/day), and chicken and poultry products (111–143 kcal/cap/day) were found to be food groups which greatly contributed to the daily energy intake.

The differences in the sources of food consumed by different respondents’ groups can be seen in Figure 1. Water/mineral water was excluded from the calculation of food sources contribution to the total food consumption. The total consumption of food, excluding water, ranged from 1213 (by school age girls) to 1568 (by adult males) g/capita/day. Adult respondents consumed homemade (567–577 g/capita/day) and street/restaurant/fast foods (502–604 g/capita/day) in a higher amount than processed foods (256–387 g/capita/day). This tendency was also found in the adolescent respondents. The school-age respondents were observed to consume the lowest amount of homemade food (291–391 g/capita/day) compared to the other respondent groups. Street/restaurant/fast foods (500–602 g/capita/day) dominated in their food consumption, followed by processed food (319–484 g/capita/day).

### 3.3. Total Daily Intake of Sugar, Salt, and Fat

The conducted food record allowed identifying 461 different food items consumed by the respondents. Supplements were excluded from the list of food groups, since their contribution to the intakes of sugar, salt, and fat was considered negligible. Data sources used to determine the sugar, salt, and fat content of all food items are shown in Table 5.

#### 3.3.1. Added Sugar Intake

Table 6 shows the results on the added sugar intake of all respondents based on different food groups. The total added sugar intake of different respondents was estimated as 34.9–45.8 g/capita/day, which is equivalent to about 9–10 teaspoons (1 teaspoon = 4 g of sucrose). The highest added sugar intake was observed in the groups of school-age boys and male adults. Beverages and snacks were found as the major contributors to the total added sugar intake of all respondents. The beverages contribution to the total added sugar intake reached 58.1% in adult males. The snacks contribution ranged from 17.2% in adolescent males to 25.7% in school-age girls. In addition, milk and dairy products were also found as another significant contributor to the added sugar intake of adolescents and school-age children.

According to the Indonesian Individual Food Consumption Survey (IFCS) 2014, the average added sugar intake of Indonesian population was estimated as 25.60 ± 23.15 g sugar/day (based on a single 24-h food recall), so it was lower than the present study findings. About 11.8% of the total population consumed >50 g sugar/day [10]. However, Atmarita et al. [11] suggested that the low level of sugar intake found in IFCS 2014 might be due to the limitation of this nationwide food consumption survey, which is also supported by the visible discrepancy between the low level of reported added sugar intake and the high prevalence of overweight and obesity. Although a single day food recall, as conducted in those surveys, allows estimating the groups mean intakes, at least two or more days of recall are needed to estimate the distribution of usual intakes in a population. Multiple 24-h recalls or validated food frequency questionnaires are considered to be more accurate methods of estimating the usual or long-term intake of total sugar in certain populations.

The total added sugar intake observed in this study was still lower than the US daily intake (2007–2010) which reached 12.5–23.5 teaspoons/day in adults (19–70 years old), 17.5–24.6 teaspoons/day in adolescents, and 14.3–21.5 teaspoons/day in children [28]. According to the Latin America Study on Nutrition and Health 2015 [29], a much higher consumption of sugar (99.4 g/day, 20.1% of total energy) was also found in the urban population of Latin American countries. The WHO recommends limiting the free sugars intake to less than 10% of total energy intake (12.5 teaspoons) to prevent dental caries and obesity, and suggests a further reduction to below 5% of total energy intake [30]. The association between excessive sugar consumption and global pandemic of obesity and cardiovascular disease was a reason for the American Heart Association to recommend a decreased free sugar intake of six teaspoons or 25 g for women and nine teaspoons or 37.5 g for men per day [31].

The collaborators of the Global Burden of Diseases (GBD), Injuries, and Risk Factors Study) Diet 2017 reported that beverages are the main contributor to the total sugar intake, with a worldwide sugar intake from sugar-sweetened beverages reaching 49 g per day, far higher than the optimal intake [7].

The percentage contribution of different food sources to the total added sugar intake is shown in Figure 2. Processed foods were identified as the main source of sugar intake for all respondents, followed by street/restaurant/fast food.

#### 3.3.2. Salt Intake

Table 7 shows the results of the salt intake of respondents based on different food groups. The total salt intake observed in this study ranged from 5.46 to 7.43 g/capita/day. The major contributors to the total salt intake in the case of all respondents were cereals and cereal products. Other food groups which also gave a significant contribution to the total salt intake included legumes and legume products (in adults), vegetable-based foods (in adults and adolescents), chicken and poultry products (in adolescents and school-age children), and snacks (in school-age children). Thout et al. [32] reported the salt consumption data from 13 countries as ranging from 6.75 to 10.7 g/capita/day (data published in 2011–2018). The WHO [33] recommends the daily salt intake to be less than 2 g of sodium per day (5 g of salt per day) in adults (≥16 years), in order to reduce blood pressure and risks of cardiovascular disease, stroke, and coronary heart disease, and the recommended intake for children (2–15 years) should be adjusted downward based on the energy requirements. In the present study, the sodium intake of respondents exceeded 2 g/day, with the highest intake found in the adolescents group (6.74 g salt/day or 2694 mg sodium/day), followed by adults (5.86 g salt/day or 2345 mg sodium/day), and children (5.82 g salt/day or 2329 mg sodium/day). In all three age groups, male subjects tended to have a higher salt intake than females. These findings were in accordance with the study of Atmarita et al. [10] who used IFCS 2014 as a reference for calculating the total salt intake. They reported a tendency of male and adolescent subjects to consume the highest amounts of salt. The total salt intake of Indonesian population reported in that study was 6.68±5.85 g/day, with about 53.7% of the population consuming more than 5 g salt/day and nearly a fifth (18.9%) of Indonesians consuming more than 10 g salt/day. However, that study did not provide the information on food sources contributing to the total salt intake of the studied population. 

Generally, about 75% of salt intake in high-income countries comes from processed foods and meals prepared outside of the home. At the same time, most of the sodium consumption in many low- and middle-income countries, especially in Asia, comes from salt added at home during cooking and at the table or through condiments such as fish sauce and soy sauce [34,35]. The present study showed that meals prepared outside of the home (street/restaurant/fast foods) were the major contributors to the total salt intake (49.8–64.6%). For adult respondents, homemade food became the second food source giving a significant contribution to the total salt intake (Figure 3), while processed foods accounted for the second largest portion of the salt intake of children (24.6%) and adolescents (20.1%). A reduction in salt intake from processed foods can be achieved by a gradual and sustained reduction in the amount of salt added to foods by the food producers/processors. The awareness-building health promoting campaigns are also very much needed to encourage consumers to use less salt during cooking at home.

#### 3.3.3. Fat Intake

The results of the fat intake of all respondents based on different food groups are presented in Table 8. The fat intake in the studied population ranged between 49.0 and 65.1 g/capita/day. The major contributors to the fat intake in the group of adult subjects were cereals and cereal products (10.6 g/capita/day or 20.2%), followed by snacks (8.34 g/capita/day or 15.9%), and chicken and poultry products (7.56 g/capita/day or 14.4%). The same tendency was also found in the group of adolescent subjects. The major contributors of fat intake in the group of school-age children were snacks (13.1 g/capita/day or 22.1%), followed by cereals and cereal products (12.7 g/capita/day or 21.4%), and chicken and poultry products (9.65 g/capita/day or 16.2%).

The percentage contribution of different food sources to the total fat intake is shown in Figure 4. Street/restaurant/fast foods appeared to be a major contributor to the fat intake of all the three subject groups. Processed foods were found as the second contributor to the fat intake of school-age children (30.7%), while homemade foods appeared to be the second contributor to the fat intake in the case of adults and adolescents (35.5% and 28.9%, respectively).

According to the joint FAO/WHO recommendations, the maximum daily fat intake should not exceed 30–35% of the total energy intake (TE), depending on the stage of growth and development (age), gender, and a number of lifestyle factors [36]. If the energy requirement is 2000 kcal/day, the maximum total fat intake should reach about 67 g/day (30% TE). The fat intake observed in this study was still below the maximum limit set by WHO. According to the study of Harika et al. [37], who summarized the data from 40 countries published in 1995–2012, the total fat intake ranged from 11.1 to 46.2% of total energy intake. The saturated fatty acids (SFA) intake was found to be positively correlated with the total fat intake (*r* = 0.76, *p* < 0.01) [37]. According to the report of the Global Burden of Diseases Nutrition and Chronic Diseases Expert Group 2014, the average global saturated fat consumption was estimated as 9.4% TE, with country-specific intakes varying dramatically (from 2.3% to 27.5% TE). The highest consumption of saturated fat in adults was found in Samoa, Kiribati, and several palm oil producing nations [38]. The dietary fat intake has been considered as one of the important dietary factors in the WHO practical advice on maintaining a healthy diet. It has been stated that excessive weight gain in adults can be reduced by maintaining the total fat intake below 30% TE. In addition, the risk of developing non-communicable diseases can be lowered through reducing saturated fats intake (<10% TE), reducing trans-fats intake (<1% TE), and replacing both saturated fats and trans-fats with unsaturated fats [39].

According to the IFCS 2014, the average total fat intake of Indonesian population was 53.3 g/capita/day, with 27% of the population consuming fat exceeding the recommended limit (>67 g/day) [10]. In addition, Hatma et al. [8] reported that the fat intake of Indonesian adults (≥18 years) from different ethnic groups (Minangkabau, Sundanese, Javanese, and Buginese) ranged from 38.6 to 64.6 g/capita/day (or equivalent to 29.5–35.8% TE), with the SFA intake ranging from 19.8% to 25.3% TE. The positive correlation between total fat intake and SFA intake was also found in that study. Although the total fat intake in the Indonesian population was generally found to be below the maximum recommended value, the quality (i.e., fatty acid composition) of the consumed fat still needs further attention. The information on the different food sources’ contribution to the fat intake is also important, so that consumers could make better choices of the origin/source of their food, and the government could develop more appropriate strategies to improve the quantity and quality of fat intake.

## 4. Conclusions

From this study we found that the total consumption of food, excluding water, ranged from 1213 (by school-age girls) to 1568 (by adult males) g/capita/day. The total added sugar consumption of the respondents was about 9-10 teaspoons per day (34.9–45.8 g/capita/day), with beverages and processed foods identified as the major contributors to the sugar intake. Although the observed values were still lower than the maximum value set by WHO, reducing the sugar intake can be associated with an improved health condition. The highest total salt intake was found in adolescent subjects (6.74 g salt/capita/day or 2694 mg sodium/capita/day), followed by adults (5.86 g salt/capita/day or 2345 mg sodium/capita/day) and school-age children (5.82 g salt/capita/day or 2330 mg sodium/capita/day). Street/restaurant/fast food greatly contributed to the total daily salt intake. The efforts to reduce salt intake still need to be undertaken in order to avoid risks of elevated blood pressure, cardiovascular disease, stroke, and coronary heart disease. The highest intake of fat was observed among the adolescents group (61.2 g/capita/day). Similar to the finding on salt intake, the major food source contributing to the fat intake was street/restaurant/fast food. Male subjects tended to consume a higher amount of salt and fat than female subjects. These findings can serve as baseline information for policy makers to improve the diet quality, especially in Indonesia. Actions towards improving the diet quality should be undertaken with a collaboration of a variety of actors throughout the food system, along with policies which target multiple food system sectors.

## Figures and Tables

**Table 1:** Subjects number by gender and age group.

Subject	Male	Female	Total
School-age children (6–12 years)	59	52	111
Adolescents (13–18 years)	51	56	107
Adults (≥19 years)	47	58	105
Total	157	166	323

**Table 2:** Health and nutritional status of adult (≥19 years) respondents.

Characteristic	Male	Female	All
Nutritional status (BMI) ^1^			
Underweight (<18.5), in %	8.5	3.4	5.7
Normal weight (18.5–24.9), in %	42.6	53.4	48.6
Overweight (25–29.9), in %	42.6	31	36.2
Obesity (>30), in %	6.4	12.1	9.5
Average BMI, mean ± SD in kg/m^2^	24.2 ± 4.3	24.9 ± 4.3	24.6 ± 4.3
Min. to max. value of BMI in kg/m^2^	15.3–34.5	11.6–33.8	11.6–34.5
Status of waist to hip ratio (WHR) ^2^			
Normal (<0.95 for male, <0.8 for female), in %	29.8	34.5	32.4
Risk (≥0.95 for male, ≥0.8 for female), in %	70.2	65.5	67.6
Average value, mean ± SD	0.9 ± 0.1	0.9 ± 0.1	0.9 ± 0.1
Min. to max. value of WHR	0.7–1.1	0.7–1.2	0.7–1.2
Blood pressure status (systole/diastole in mmHg) ^3^			
Normal (<120/<80), in %	55.3	70.7	63.8
Pre-hypertension (120–129/<80), in %	31.9	20.7	25.7
Hypertension stage 1 (130–139/80–89), in %	10.6	6.9	8.6
Hypertension stage 2 (≥140/≥90), in %	2.1	1.7	1.9
Diabetes Mellitus (DM) history of family member			
Yes, in %	25.5	32.8	29.5
No, in %	74.5	67.2	70.5
Hypertension history of family member			
Yes, in %	21.3	37.9	30.5
No, in %	78.7	62.1	69.5
Cardiovascular (CVD) disease history of family member			
Yes, in %	21.3	12.1	11.4
No, in %	89.4	87.9	88.6
Suffering from DM			
Yes, in %	0	3.4	1.9
No, in %	100	96.6	98.1
Suffering from hypertension			
Yes, in %	0	3.4	3.8
No, in %	95.7	96.6	96.2
Suffering from CVD			
Yes, in %	0	0	0
No, in %	100	100	100

^1^ Categorization of nutritional status (BMI) was based on the World Health Organization (WHO) [24]; ^2^ categorization of waist to hip ratio status was based on the WHO [25]; ^3^ categorization of blood pressure status was based on the American Heart Association (AHA) [26].

**Table 3:** Health and nutritional status of adolescents and school-age respondents.

Characteristic	Adolescents (13–18 Years Old)			School-Age Children (6–12 Years Old)		
	Male	Female	All	Male	Female	All
Body height status ^1^ by age, in %						
Stunted	9.8	1.8	5.6	5.1	11.5	8.1
Normal	90.2	98.2	94.4	89.8	88.5	89.2
Tall	0	0	0	5.1	0	0
BMI status by age ^1^, in %						
Underweight	13.7	7.2	10.2	6.8	5.8	6.3
Normal	66.7	83.9	75.7	76.3	84.6	80.2
Overweight	19.6	5.4	12.1	16.9	9.6	13.5
Obese	0	3.6	1.9	0	0	0
Blood pressure status ^2^, in %						
Normal	90.2	98.2	94.4	98.3	98.1	98.2
Pre-hypertension	7.8	0	3.7	1.7	1.9	1.8
Hypertension	2	1.8	1.9	0	0	0
Diabetes Mellitus (DM) history of family member						
Yes, in %	15.7	17.9	16.8	22	23.1	22.5
No, in %	84.3	82.1	83.2	78	76.9	77.5
Hypertension history of family member						
Yes, in %	15.7	23.2	19.6	23.7	26.9	25.2
No, in %	84.3	76.8	94.4	76.3	73.1	74.8
Cardiovascular (CVD) disease history of family member						
Yes, in %	3.9	7.1	5.6	10.2	11.5	10.8
No, in %	96.1	92.9	94.4	89.8	88.5	89.2
Suffering from DM						
Yes, in %	0	0	0	0	0	0
No, in %	100	100	100	100	100	100
Suffering from hypertension						
Yes, in %	3.9	0	1.9	0	0	0
No, in %	96.1	100	98.1	100	100	100
Suffering from CVD						
Yes, in %	0	0	0	0	0	0
No, in %	100	100	100	100	100	100

^1^ Categorization of body height and BMI status was based on the WHO growth reference [27]; ^2^ categorization of blood pressure status was based on the AHA [26].

**Table 4:** Food consumption pattern of different respondents groups.

No	Food Group	Food Consumption (g/capita/day)								
		Adults (Mean ± SD)			Adolescents (Mean ± SD)			School-Age Group (Mean ± SD)		
		Male	Female	All	Male	Female	All	Male	Female	All
1	Bakery products	18.5 ± 32.8	15.5 ± 29.1	16.9 ± 30.7	18.4 ± 31.2	32.5 ± 111	25.7 ± 82.9	19.0 ± 30.5	21 ± 33.1	19.9 ± 31.6
2	Beverages	1186 ± 612	1215 ± 669	1202 ± 644	1077 ± 692	941 ± 444	1006 ± 577	1055 ± 665	889 ± 442	977 ± 575
3	Cereals and cereal products	635 ± 235	523 ± 216	573 ± 231	704 ± 285	552 ± 181	624 ± 247	563 ± 253	504 ± 157	535 ± 214
4	Eggs and egg products	35.1 ± 44.8	15.6 ± 20.0	24.3 ± 34.7	28.4 ± 41.4	21.4 ± 24.3	24.8 ± 33.6	30.3 ± 33.2	42.3 ± 39.9	35.9 ± 36.8
5	Fish and fish products (incl. seafood)	34.8 ± 50.2	25.0 ± 46.0	29.4 ± 47.9	28.0 ± 47.3	19.5 ± 29.1	23.5 ± 38.9	16.9 ± 28.2	17.5 ± 30.7	17.2 ± 29.3
6	Fruits and fruit products	75 ± 159	55.6 ± 76.8	64.3 ± 121	37.1 ± 66.8	35.1 ± 71.4	36.0 ± 68.9	14.9 ± 35.5	31.5 ± 63.3	22.7 ± 50.7
7	Legumes and legume products	53.2 ± 64.3	52.1 ± 91.5	52.6 ± 80.1	22.7 ± 33.4	28.8 ± 41.3	25.9 ± 37.7	34.9 ± 81.5	20.0 ± 45.5	27.9 ± 67.2
8	Meat and meat products	20 ± 41.0	25.8 ± 69.0	23.2 ± 58.2	22.0 ± 56	23.8 ± 45.1	23 ± 50.3	17.6 ± 47.1	8.8 ± 21.1	13.5 ± 37.3
9	Milk and dairy products	22.5 ± 70.5	16.8 ± 58.7	19.4 ± 64	84.4 ± 124	101 ± 170.5	93 ± 150	186 ± 167	139 ± 185	164 ± 176
10	Chicken and poultry products	55.7 ± 69.7	57.7 ± 77.2	56.8 ± 73.6	62.5 ± 68.9	60.9 ± 71.5	61.6 ± 69.9	71.4 ± 71.8	60.0 ± 57.1	66.1 ± 65.3
11	Snacks	75.1 ± 99.1	81.7 ± 81.7	78.7 ± 88.6	57.1 ± 70.4	76.4 ± 91.6	67.2 ± 82.4	96.3 ± 109	76.7 ± 83.9	87.1 ± 98.2
12	Vegetables and vegetable products	124 ± 96.7	111 ± 97.2	117 ± 96.8	80.6 ± 101	68 ± 68	74 ± 85.3	65.3 ± 60.4	58.0 ± 59.2	61.9 ± 59.7
13	Supplements	0	0	0	0.0 ± 0.2	0.0 ± 0.1	0.0 ± 0.1	0	0	0
Total		2334	2195	2257	2222	1960	2084	2169	1868	2028

**Table 5:** Data sources for determining the sugar, salt, and fat content.

Data Sources	Fat Content		Sugar Content		Salt Content	
	Number of Food Items	%	Number of Food Items	%	Number of Food Items	%
Food composition databases ^1^	37	8.03	8	1.74	1	0.22
Recipe elaboration	291	63.12	249	54.01	227	49.24
Food labels ^2^	109	23.64	115	24.95	84	18.22
Laboratory analysis	0	0.00	22	4.77	40	8.68
Assigned as zero	4	0.87	67	14.53	109	23.64
Unavailable data	20	4.34	0	0.00	0	0.00
Total	461	100	461	100	461	100

^1^ The Indonesian Food Composition Table 2005 [11]; the USDA National Nutrient Database for Standard Reference 2015 [12]; the Food Composition Table of Singapore [13]; ^2^ commercial products enlisted by the National Agency for Drug and Food Control—Indonesia.

**Table 6:** Added sugar intake among the respondents based on different food groups.

No.	Food Group	Added Sugar Intake (g/capita/day) ^1^								
		Adults			Adolescents			School-Age Children		
		Male	Female	All	Male	Female	All	Male	Female	All
1	Bakery products	1.42 (0.28–13.5) ^2^	0.86 (0.29–11.7)	1.11 (0.28–13.5)	1.11 (0.52–7.32)	1.45 (0.10–28.2)	1.29 (0.10–28.2)	1.63 (0.40–12.1)	1.13 (0.52–20.3)	1.40 (0.40–20.3)
2	Beverages	25.9 (0–92.04)	23.4 (0.10–101.9)	24.5 (0–101.9)	18.2 (0.11–81.1)	19.4 (0.10–71.1)	18.8 (0.10–81.1)	17.1 (0–68.6)	13.4 (0.21–51.5)	15.4 (0–68.6)
3	Cereals and cereal products	3.37 (0–45.3)	2.36 (0–17.1)	2.81 (0–45.3)	3.55 (0–15.5)	3.84 (0–38.7)	3.70 (0–38.7)	3.88 (0–17.9)	2.29 (0–9.00)	3.14 (0–17.9)
4	Eggs and egg products	0.23 (0–1.24)	0.08 (0–0.77)	0.15 (0–1.24)	0.21 (0–1.99)	0.14 (0–0.83)	0.18 (0–1.99)	0.14 (0–0.687)	0.23 (0–2.85)	0.18 (0–2.85)
5	Fish and fish products (incl. seafood)	0.16 (0–1.49)	0.17 (0–2.04)	0.17 (0–2.04)	0.757 (0.000–16.562)	0.114 (0.000–0.780)	0.421 (0.000–16.562)	0.085 (0.000–1.115)	0.092 (0.000–0.884)	0.088 (0.000–1.115)
6	Fruits and fruit products	0	0.52 (0–14.3)	0.29 (0–14.3)	1.23 (0–22.0)	0.27 (0–3.67)	0.73 (0–22.0)	1.88 (0–30.6)	1.13 (0–19.5)	1.53 (0–30.6)
7	Legumes and legume products	1.43 (0–11.04)	1.89 (0–48.5)	1.68 (0–48.5)	1.33 (0–22.0)	0.63 (0–16.2)	0.96 (0–22.0)	2.64 (0–43.2)	1.18 (0–19.5)	1.96 (0–43.2)
8	Meat and meat products	0.10 (0–1.48)	0.21 (0–8.21)	0.16 (0–8.21)	0.13 (0–3.80)	0.30 (0–3.80)	0.22 (0–3.80)	0.18 (0–3.75)	0.06 (0–1.59)	0.12 (0–3.75)
9	Milk and dairy products	0.70 (0–15.5)	2.24 (0–94.5)	1.55 (0–94.5)	4.39 (0–27.0)	5.15 (0–35.5)	4.79 (0–35.5)	8.30 (0–30.20)	6.42 (0–38.3)	7.42 (0–38.3)
10	Chicken and poultry products	0.42 (0–2.73)	0.47 (0–3.80)	0.45 (0–3.80)	0.45 (0–2.72)	0.49 (0–4.93)	0.48 (0–4.93)	0.40 (0–3.17)	0.48 (0–6.03)	0.44 (0–6.03)
11	Snacks	9.84 (0–129)	9.59 (0–48.2)	9.71 (0–129)	6.58 (0–60.9)	7.9 (0–35.35)	7.28 (0–60.9)	10.7 (0–58.6)	8.95 (0–66.8)	9.88 (0–66.78)
12	Vegetables and vegetable products	1.00 (0–6.03)	1.31 (0–12.2)	1.17 (0–12.2)	1.38 (0.03–10.6)	0.73 (0–3.16)	1.04 (0–10.6)	0.54 (0–2.86)	0.44 (0–5.72)	0.49 (0–5.72)
Total		44.6a ^3^	43.1a	43.8A ^4^	38.3a	40.3a	39.3A	45.8a	34.9b	40.7A

^1^ Added sugar intake was expressed as the mean value in g/capita/day; ^2^ the values in the parentheses indicate the minimum and maximum value observed in this study (min-max); ^3^ different lower case letters within the last row indicate significant differences between male and female respondents within each age group, determined by the independent samples *t*-test (*p*-value < 0.05); ^4^ different capital letters within the last row indicate significant differences between different age groups (*p*-value < 0.05).

**Table 7:** Salt intake among the respondents based on different food groups.

No.	Food Group	Salt Intake (g/capita/day) ^1^								
		Adults			Adolescents			School-Age Children		
		Male	Female	All	Male	Female	All	Male	Female	All
1	Bakery products	0.18 (0–1.54) ^2^	0.15 (0–1.52)	0.16 (0–1.54)	0.17 (0.08–1.16)	0.14 (0.02–1.04)	0.16 (0.02–1.16)	0.20 (0–2.86)	0.15 (0–1.52)	0.17 (0–2.86)
2	Beverages	0.13 (0–1.49)	0.04 (0–0.35)	0.08 (0–1.49)	0.16 (0–1.20)	0.13 (0–1.05)	0.15 (0–1.20)	0.02 (0–0.23)	0.04 (0–0.35)	0.02 (0–0.23)
3	Cereals and cereal products	2.23 (0–6.91)	2.09 (0–10.5)	2.15 (0–10.5)	3.48 (0–9.23)	2.78 (0–9.62)	3.11 (0–9.62)	2.23 (0–6.73)	2.09 (0–10.52)	2.46 (0.000–7.51)
4	Eggs and egg products	0.52 (0.10–3.42)	0.21 (0–1.56)	0.35 (0–3.42)	0.45 (0.06–2.96)	0.33 (0.08–2.01)	0.39 (0.06–2.92)	0.60 (0–2.58)	0.21 (0–1.56)	0.51 (0–2.58)
5	Fish and fish products (incl. seafood)	0.74 (0.04–7.60)	0.41 (0.02–5.38)	0.56 (0.02–7.60)	0.54 (0.07–4.29)	0.29 (0.05–3.83)	0.41 (0.05–4.29)	0.37 (0.06–3.60)	0.41 (0.02–5.38)	0.35 (0.06–3.60)
6	Fruits and fruit products	0.003 (0–0.13)	0.03 (0–0.91)	0.02 (0–0.91)	0.0003 (0–0.01)	0.01 (0–0.35)	0.004 (0–0.35)	0.01 (0–0.50)	0.03 (0–0.91)	0.01 (0–0.50)
7	Legumes and legume products	0.76 (0.17–2.89)	0.60 (0.001–2.94)	0.67 (0.001–2.94)	0.42 (0.04–2.60)	0.39 (0–2.61)	0.41 (0–2.61)	0.28 (0.001–3.22)	0.60 (0.001–2.94)	0.31 (0–3.22)
8	Meat and meat products	0.12 (0.02–1.20)	0.16 (0.05–1.77)	0.14 (0.02–1.77)	0.21 (0.03–2.34)	0.22 (0.07–1.92)	0.22 (0.03–2.34)	0.07 (0.06–0.62)	0.16 (0.05–1.77)	0.15 (0.06–2.53)
9	Milk and dairy products	0.02 (0–0.46)	0.01 (0–0.34)	0.01 (0–0.46)	0.15 (0.04–0.80)	0.18 (0–1.40)	0.16 (0–1.40)	0.01 (0–0.14)	0.01 (0–0.34)	0.01 (0–0.14)
10	Chicken and poultry products	0.60 (0.12–3.63)	0.56 (0.11–4.20)	0.58 (0.11–4.20)	0.70 (0.02–4.03)	0.62 (0.10–3.38)	0.66 (0.02–4.03)	0.69 (0.13–2.27)	0.56 (0.11–4.20)	0.70 (0.05–2.75)
11	Snacks	0.44 (0–2.28)	0.51 (0–4.06)	0.48 (0–4.06)	0.48 (0–3.35)	0.54 (0.04–2.18)	0.51 (0–3.35)	0.53 (0–4.08)	0.51 (0–4.06)	0.61 (0–4.15)
12	Vegetables and vegetable products	0.62 (0–1.84)	0.69 (0.06–2.55)	0.66 (0–2.55)	0.66 (0.01–5.18)	0.48 (0–2.79)	0.56 (0–5.18)	0.59 (0–4.03)	0.69 (0.06–2.55)	0.54 (0–5.22)
Total		6.36a ^3^	5.46a	5.86B ^4^	7.43a	6.11b	6.74A	6.02a	5.60a	5.83B

^1^ Salt intake was expressed as the mean value in g/capita/day; ^2^ the values in the parentheses indicate the minimum and maximum value observed in this study (min-max); ^3^ different lower case letters within the last row indicate significant differences between male and female respondents within each age group, determined by the independent samples *t*-test (*p*-value < 0.05); ^4^ different capital letters within the last row indicate significant differences between different age groups (*p*-value < 0.05).

**Table 8:** Fat intake among the respondents based on different food groups.

No.	Food Group	Fat Intake (g/capita/day) ^1^								
		Adults			Adolescents			School-Age Children		
		Male	Female	All	Male	Female	All	Male	Female	All
1	Bakery products	2.04 (0.28–17.6) ^2^	1.96 (0.71–25.4)	1.99 (0.28–25.4)	1.98 (1.62–19.3)	2.57 (1.74–25.4)	2.30 (1.62–25.4)	2.63 (0.89–4.15)	4.15 (0.63–30.6)	3.34 (0.63–30.6)
2	Beverages	5.33 (0–75.3)	3.16 (0–86.6)	4.13 (0–86.6)	0.96 (0–19.8)	2.50 (0–61.9)	1.77 (0–61.9)	3.00 (0–61.9)	1.00 (0–11.4)	2.06 (0–61.9)
3	Cereals and cereal products	11.7 (0.54–53.9)	9.69 (0.41–30.5)	10.59 (0.41–53.99)	23.56 (0–0.59)	16.3 (0.47–79.6)	19.8 (0.47–86.3)	13.6 (0.51–56.1)	11.7 (0.55–42.2)	12.7 (0.51–56.1)
4	Eggs and egg products	5.01 (2.64–19.60)	2.52 (2.07–13.3)	3.63 (2.07–19.6)	4.23 (1.93–16.4)	3.28 (1.88–17.6)	3.73 (1.88–17.6)	4.54 (1.76–20.4)	6.19 (2.62–25.90)	5.31 (1.76–25.90)
5	Fish and fish products (incl. seafood)	4.74 (0.86–34.3)	3.50 (0.61–25.8)	4.05 (0.61–34.3)	3.31 (1.26–26.4)	2.60 (0.48–18.5)	2.94 (0.48–26.37)	2.84 (0.61–18.69)	2.52 (0.73–28.2)	2.69 (0.61–28.2)
6	Fruits and fruit products	0.23 (0.02–2.76)	0.49 (0–8.70)	0.37 (0–8.70)	0.11 (0–1.03)	0.20 (0–7.04)	0.16 (0–7.04)	0.18 (0–4.98)	0.22 (0–6.99)	0.20 (0–6.99)
7	Legumes and legume products	5.93 (1.50–35.1)	4.08 (0–28.8)	4.91 (0–35.1)	2.49 (0.01–25.5)	2.91 (0–15.8)	2.71 (0–25.50)	2.14 (0–13.4)	2.20 (0–44.5)	2.17 (0–44.5)
8	Meat and meat products	3.61 (0.60–45.4)	3.15 (0.37–72.7)	3.36 (0.37–72.7)	3.84 (0.37–51.2)	2.65 (0.08–53.7)	3.22 (0.08–53.7)	2.05 (1.33–33.6)	1.85 (0.77–36.6)	1.96 (0.77–36.6)
9	Milk and dairy products	0.25 (0–3.57)	0.44 (0.91–14.6)	0.35 (0–14.58)	1.88 (0.88–10.4)	2.87 (0.15–21.3)	2.40 (0.15–21.3)	4.89 (0–18.13)	3.28 (0.07–18.00)	4.13 (0–18.13)
10	Chicken and poultry products	7.60 (2.01–33.6)	7.52 (1.81–57.2)	7.56 (1.81–57.2)	9.44 (3.42–48.9)	9.83 (2.85–42.1)	9.65 (2.85–48.9)	9.79 (2.05–51.5)	9.50 (2.09–43.0)	9.65 (2.05–51.5)
11	Snacks	7.36 (0.21–30.3)	9.13 (0.04–8.34)	8.34 (0.04–46.9)	10.3 (0.92–43.7)	9.81 (0.49–62.92)	10.0 (0.49–62.9)	14.8 (0.095–148)	11.3 (0.37–64.7)	13.1 (0.095–148)
12	Vegetables and vegetable products	3.12 (0.02–8.35)	3.39 (0.02–19.2)	3.26 (0.02–19.2)	3.01 (0–26.2)	2.13 (0.01–12.4)	2.55 (0–26.2)	2.02 (0–9.16)	2.13 (0.13–10.4)	2.07 (0–10.4)
	Total	56.9a ^3^	49.0a	52.6B ^4^	65.1a	57.7a	61.2A	62.5a	56.0a	59.4A

^1^ Fat intake was expressed as the mean value in g/capita/day; ^2^ the values in the parentheses indicate the minimum and maximum value observed in this study (min-max); ^3^ different lower case letters within the last row indicate significant differences between male and female respondents within each age group, determined by the independent samples *t*-test (*p*-value < 0.05); ^4^ different capital letters within the last row indicate significant differences between different age groups (*p*-value < 0.05).

## Data Availability

Data will be made available upon request, by author Nuri Andarwulan.

## Supplementary Materials

The following are available online at https://www.mdpi.com/article/10.3390/nu13041289/s1. Table S1: Calorie intake among the respondents (total and from different food groups).
