# Supplementary material for: Food Consumption Pattern and the Intake of Sugar, Salt, and Fat in the South Jakarta City—Indonesia
Source: Nutrients. 2021 Apr 14;13(4):1289. doi: 10.3390/nu13041289 (PMC8070674; doi:10.3390/nu13041289)
Supplement: Supplementary file 1 [file nutrients-13-01289-s001.pdf]

Table S1. Calorie intake among the respondents (total and from different food groups).

No	Food Group	Calorie intake (kcal/cap/day)[1]								
		**Adults (Mean±SD)**			**Adolescents (Mean±SD)**			**School-age children (Mean±SD)**		
		**Male**	**Female**	**All**	**Male**	**Female**	**All**	**Male**	**Female**	**All**
1	Bakery products	61±90	53±119	57±107	60±90	68±128	64±111	67±109	81±147	73±128
2	Beverages	212±194	167±225	187±210	92±83	143±154	119±128	148±190	103±108	127±159
3	Cereals and cereal products	853±330	683±280	759±314	1044±436	762±274	897±386	781±346	698±250	742±306
4	Eggs and egg products	62±54	31±28	45±45	53±55	41±44	47±50	58±51	78±58	67±56
5	Fish and fish products	77±125	48±89	61±108	48±71	35±61	42±68	37±58	37±80	37±69
6	Fruits and fruit products	42±117	42±66	42±91	22±42	20±50	21±45	12±46	24±65	17±58
7	Legumes and legume products	89±108	65±97	76±102	43±72	45±70	44±70	45±81	36±121	41±97
8	Meat and meat products	42±137	45±211	44±180	49±180	38±139	43±159	28±96	21±105	25±98
9	Milk and dairy products	13±56	15±107	14±84	63±119	77±126	70±122	125±108	96±105	111±106
10	Chicken and poultry products	114±96	109±142	111±123	139±144	146±167	143±156	145±117	140±111	142±115
11	Snacks	192±241	215±181	205±207	205±228	205±216	205±221	278±261	246±257	263±259
12	Vegetables and vegetable products	96±84	106±132	101±113	81±136	56±65	68±106	59±62	58±79	58±70
	**Total**	**1854±491a[2]**	**1579±537b**	**1702±533A[3]**	**1901±651a**	**1636±494a**	**1762±586A**	**1781±591a**	**1618±493a**	**1705±551A**
	**Min-Max value**	**743-2917**	**648-2920**	**648-2920**	**941-4176**	**682-2692**	**682-4176**	**1047-3915**	**749-3046**	**749-3915**
	**Percentile 75**	**2241**	**1889**	**2054**	**2296**	**1978**	**2090**	**2052**	**1757**	**1944**

[1] Calorie intake was expressed as mean **± SD** in kcal/capita/day; [2] Different lower case letters within the total calorie intake row indicate significant differences between male and female respondents within each age group, determined by independent samples t-test (p-value < 0.05); [3] Different capital letters within the total calorie intake row indicate significant differences between different age groups (p-value < 0.05).
